# Unlocking the Fluorine‐Free Buoy Effect: Surface‐Enriched Ruthenium Polypyridine Complexes in Ionic Liquids

**DOI:** 10.1002/open.202400092

**Published:** 2024-04-30

**Authors:** Luciano Sanchez Merlinsky, Daniel Hemmeter, Luis M. Baraldo, Florian Maier, Hans‐Peter Steinrück, Federico J. Williams

**Affiliations:** ^1^ Departamento de Química Inorgánica Analítica y Química Física Facultad de Ciencias Exactas y Naturales Universidad de Buenos Aires Buenos Aires Argentina; ^2^ Instituto de Química Física de los Materiales Medio Ambiente y Energía CONICET-Universidad de Buenos Aires Buenos Aires Argentina; ^3^ Lehrstuhl für Physikalische Chemie II Friedrich-Alexander-Universität Erlangen-Nürnberg Egerlandstraße 3 Erlangen Germany

## Abstract

Controlling the local concentration of metal complexes at the surface of ionic liquids (ILs) is a highly sought‐after objective due to its pivotal implications in supported ionic liquid phase (SILP) catalysis. Equally important is to avoid per‐ and polyfluorinated substances due to environmental concerns. Herein, we investigate the surface enrichment of Ru polypyridyl complexes with fluorine‐free alkylic side groups of varying lengths and shapes, using the hydrophilic IL [C_2_C_1_Im][OAc] as solvent. Additional charged carboxylate groups are included into the polypyridyl ligands to increase the solubility of the complex in the IL. When the ligand system is functionalized with long and hydrophobic alkyl side chains, the complex predominantly localizes at the IL/vacuum interface, as deduced from angle‐resolved X‐ray photoelectron spectroscopy. Conversely, in the presence of short or more bulky substituents, no surface enrichment is observed. This buoy‐like behaviour with fluorine‐free side groups is explored for 0.05 %_mol_ to 1 %_mol_ solutions. Intriguingly, surface saturation occurs at approximately 0.5 %_mol_, which is beneficial to the efficient operation of catalytic systems featuring high surface areas, such as SILP catalysts.

## Graphical abstract

Functionalizing metal complex ligands with long, fluorine‐free alkyl side chains, induces pronounced enrichment at the IL/vacuum interface, as confirmed by Angle‐resolved X‐ray Photoelectron Spectroscopy.

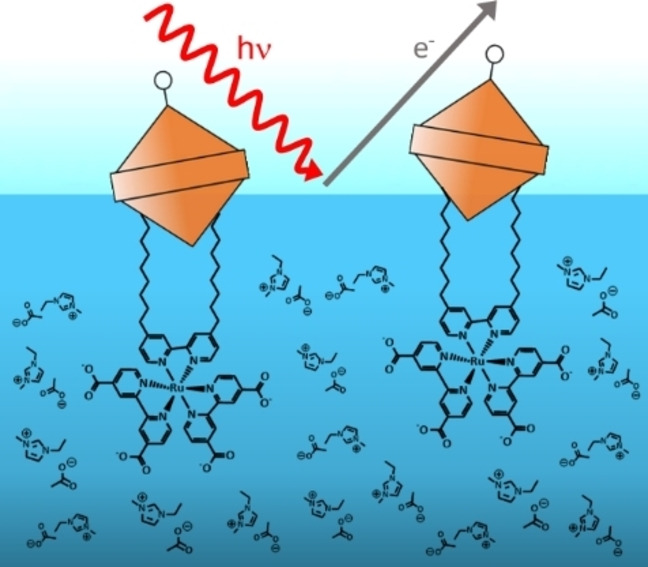

## Introduction

Ionic liquids (ILs) have recently found applications as alternative solvents in numerous transition‐metal‐catalysed reactions due to their extremely low volatility, non‐flammability, thermal stability, and wide‐ranging tailorable properties.^[1]^ A particularly crucial application is in supported ionic liquid phase (SILP) catalysis, where IL thin films containing dissolved metal catalysts impregnate high‐surface area supports.^[2]^ These macroscopically solid systems combine the advantages of heterogeneous catalysis, enabling efficient separation of products and catalysts, with the benefits of homogeneous catalysis, conferring high selectivity. SILP catalysts exhibit promising performance in many gas‐phase reactions of industrial relevance.^[3]^ In many cases, they operate under milder conditions and show higher selectivity than their heterogeneous counterparts.^[4]^ SILP systems containing Ru complexes can catalyse important reactions such as methanol reforming,^[5]^ the water‐gas shift reaction,^[6]^ CO_2_ hydrogenation,^[7]^ and the alkoxy carbonylation of olefins with CO_2_.^[8]^ Furthermore, Ru polypyridyl complexes dissolved in ILs are promising candidates to reduce carbon dioxide,^[9]^ or in the construction of stable and efficient dye‐sensitized solar cells.^[10]^

Catalytic reactions in SILP systems require that the reactants enter the IL film from the gas phase, diffuse towards the catalytically active species, react to form products, which diffuse out of the IL phase.^[11]^ The processes of dissolution and diffusion in the IL phase can pose significant limitations to the performance of SILP catalysts. Therefore, a preferential placement of the catalytically active complex right at the IL/gas interface could minimize transport barriers enhancing the overall catalytic efficiency. Thus, an ideal SILP catalyst should promote surface enrichment of the dissolved metallic complex at the IL/gas interface.

Over the past decades, much research has been carried out to understand surfaces of neat ILs or mixture of ILs.^[12]^ However, the number of surface investigations on IL films containing dissolved metal complexes is much lower. Different studies indicate that the chemical nature of the ligands influences the local concentration of the metal complex at the IL/vacuum interface.^[13]^ For example, functionalization of [Rh(acac)(CO)_2_] with the trisodium 3,3′,3′′‐phosphanetriyltri(benzene‐1‐sulfonate) (TPPTS) ligand resulted in surface enrichment,^[13a]^ whereas Pt(II) and Pd(II) complexes with ligand systems derived from nitrile‐functionalized ionic liquids showed homogeneous distribution of the complexes in the IL with no enrichment in the outermost surface layers.^[13c]^ Notably, adding perfluorinated substituents to Pt(II) complexes resulted in surface enrichment, while without these substituents no such effect could be observed.[^13d^, ^13e^] However, the former suffer from the environmental problems associated with per‐ and polyfluorinated substances (PFAS).^[14]^ Therefore, it is important to continue exploring and tailoring ligands for transition metals that promote surface enrichment and are free of polyfluorinated alkyl groups.

Previous studies have shown that adding surfactants or surface‐active contaminations (e. g. polysiloxanes) with long hydrophobic tails in ILs can lead to the formation of a surfactant layer at the liquid/gas interface with their non‐polar tails directed towards the gas phase forming the typical monolayer that is usually seen in analogous aqueous systems, accompanied by a decrease in surface tension.^[15]^ Moreover, a series of metallosurfactants has been investigated in aqueous media under ambient conditions.^[16]^ For example, [Ru(bipyridine)_3_]^2+^ complexes with alkyl side chains between 12 and 19 carbon atoms attached to one of the bidentate ligands have shown to be surface‐active, with different micelle shapes, adsorption rates and structures at the water/air interface depending on the length, position and number of side chains in the molecule.[^16b^, ^16c^, ^16d^, ^16e^]

Recently for ILs, a first example of surface enrichment was reported for a metallosurfactant‐type Ru complex with a tri‐octyl phosphine ligand when dissolved in [C_2_C_1_Im][Tf_2_N] as based on non‐reactive atom scattering with an hyperthermal F‐Atoms probe, X‐ray photoelectron spectroscopy and time‐of‐flight secondary ion mass spectrometry.^[13b]^ This study was, however, performed for only one type of complex and only for one single complex concentration, and with a limited XPS surface sensitivity of ~7 nm; moreover, the solution was also contaminated with a highly surface‐active polysiloxane, which might had an influence on the surface enrichment of the complex.^[13b]^

**Figure 1:**
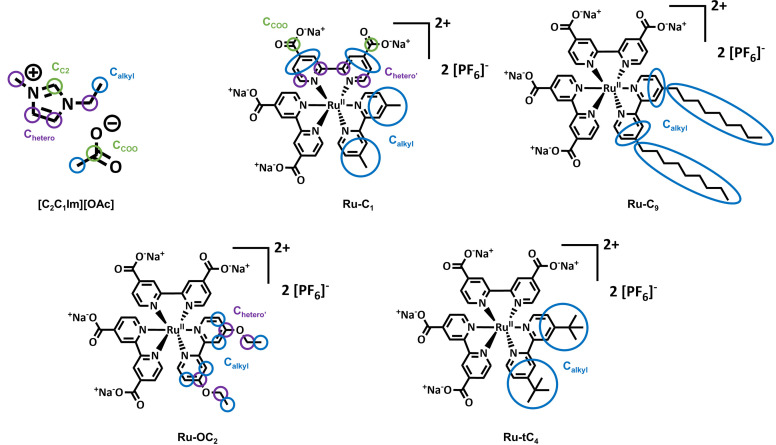
Molecular structures of the complexes and the IL employed in this work with the corresponding assignment of carbon species to the deconvolved XPS signals. The complexes were synthesized as Na^+^ and [PF_6_]^−^ salts in all cases.

In order to obtain a more detailed understanding, we herein focus on evaluating routes to tailor the surface enrichment of PFA‐free Ru complexes in ionic liquids. For this purpose, we have designed Ru polypyridyl complexes with alkyl chains of varying lengths and shapes to investigate surface enrichment at the IL/vacuum interface using angle‐resolved X‐ray Photoelectron Spectroscopy (ARXPS) under ultraclean vacuum conditions. We performed our measurements at 0° (bulk‐sensitive) and 80° (sensitive to the topmost layer) emission angle (see below). Specifically, we examined the behaviour of Ru complexes with bipyridine ligands functionalized with four carboxylate side groups, creating a highly polar headgroup to enhance their solubilities. We introduced two methyl (Ru‐C_1_), ethoxy (Ru‐C_2_), tert‐butyl (Ru‐tC_4_) or n‐nonyl (Ru‐C_9_) side chains to control the surface composition in 1‐ethyl‐3‐methylimidazolium acetate [C_2_C_1_Im][OAc] (Figure 1), which is highly hydrophilic and has a high surface tension.^[17]^ Our findings demonstrate that the non‐fluorinated, hydrophobic nonyl chains induce a strong enrichment of the complex at the surface of the solution, similar to a comparable metallosurfactant Ru polypyridyl complex in aqueous solution.[^16b^, ^16c^, ^16d^, ^16e^] Conversely, we find no such enrichment for a complex with shorter alkyl chains or with bulky hydrophobic tert‐butyl groups.

## Experimental Methods

Experimental details, such as syntheses, sample preparation and information on ARXPS analyses can be found in the supporting information (SI).

## Results and Discussion

ARXPS is a powerful technique for characterizing the near‐surface region of ILs in detail, providing information on chemical state and compositional depth‐distributions.^[18]^ When using Al Kα radiation, electron emission normal to the surface (at 0°) yields information on the top 6–9 nm of organic films, which typically reflects the bulk composition. On the other hand, grazing electron emission (at 80°) offers insights into the top 1–1.5 nm, mainly capturing details of the topmost molecular layer.^[19]^

**Figure 2:**
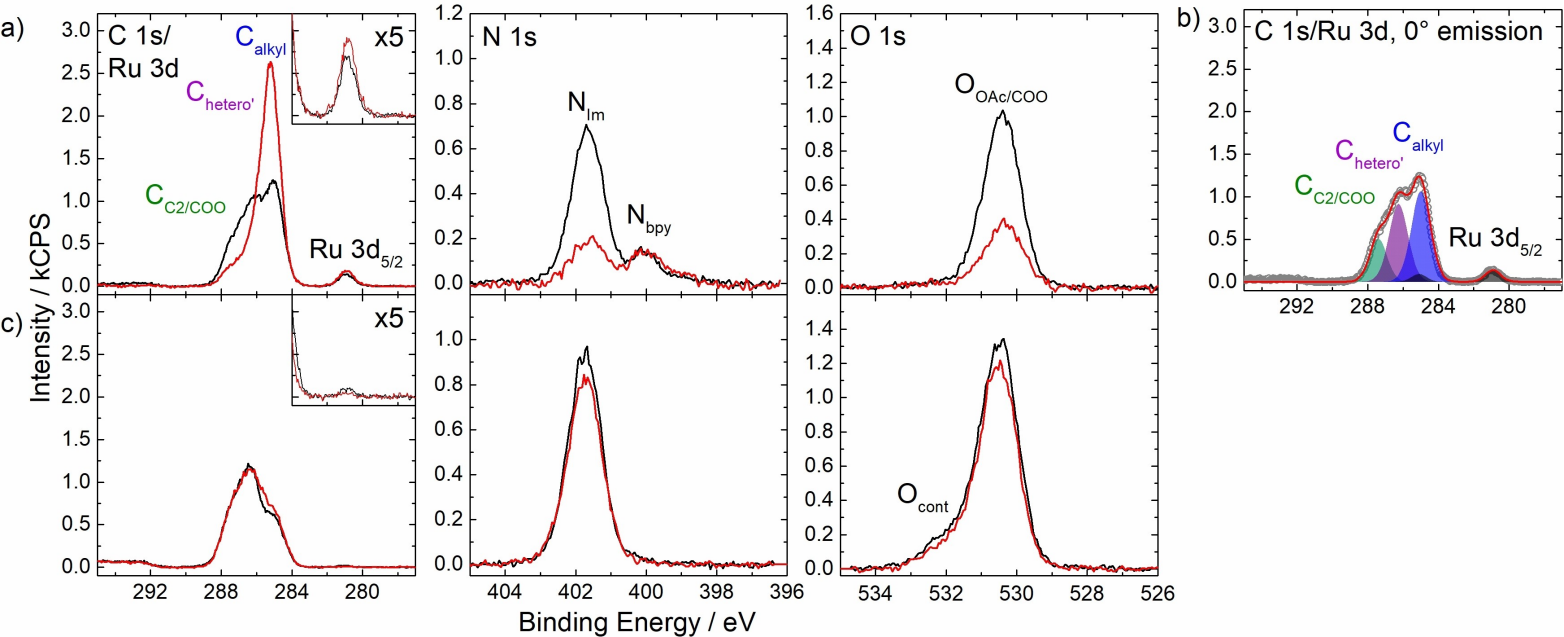
C 1s/Ru 3d, N 1s and O 1s XPS spectra regions of 1 %_mol_ solutions of a) Ru‐C_9_ (top row) and c) Ru‐C_1_ (bottom row) in [C_2_C_1_Im][OAc] at 0° (black) and 80° (red) emission. Upscaled Ru 3d_5/2_ signals (x5) are depicted in the insets. b) Fitting of the C 1s/Ru 3d XPS spectra region of the Ru‐C_9_ solution at 0° emission and assignment of peaks to the molecular structure (cf. Figure 1). In the O 1s region of the solution of Ru‐C_1_ a small amount of a non‐surface‐active contamination O_cont_ from the synthesis procedure was also identified which is not expected to affect the surface structure.

Figure 2a shows the C 1s/Ru 3d, N 1s and O 1s regions measured for a 1 %_mol_ Ru‐C_9_ solution in [C_2_C_1_Im][OAc] at 0° (black) and 80° (red) emission. Note that the full set of spectra is displayed in Figure S1 in the SI, and the quantitative analysis of the binding energies and peak intensities is provided in Table S1a. In the C 1s/Ru 3d region, a broad signal envelope centred at 286 eV is observed, which corresponds to the C atoms of the complex and the IL; it is typically deconvolved using three contributions:[^19^, ^20^] the carboxylate groups and the C_2_ imidazolium carbon were fitted as one signal C_C2/COO_ at 287.4 eV, the carbon atoms bound to one heteroatom as C_hetero’_ at 286.3 eV, and the alkylic carbon atoms as C_alkyl_ at 285.0 eV. Figure 2b illustrates the fitting employed at 0° emission; for assignment of the peaks to the molecular structures, see Figure 1. The Ru 3d_5/2_ signal at 280.9 eV is consistent with an oxidation state of +2.^[20]^ Note that the Ru 3d_3/2_ peak at 285.1 eV is hidden under the prominent C_alkyl_ signal (Figure 2b). In the N 1s region, the peak at 401.7 eV is assigned to the imidazolium nitrogen atoms from the IL and the signal at 400.0 eV to the bipyridine ligands from the complex.^[20]^ The single O 1s signal at 530.4 eV stems from the [OAc]^−^ anion of the IL and the COO^−^ groups of the complex. The Na 1s signal at 1070.8 eV originates from the dissolved Na^+^ counterions of the carboxylate groups (see Figure S1). Interestingly, no F 1s and P 2p signals from the [PF_6_]^−^ counterions of the complex are detected, which indicates a strong surface depletion of these anions in solution. Notably, XPS of solid Ru‐C_9_ confirmed the presence of [PF_6_]^−^ in the compound (see Figure S2). Concerning the absence of the [PF_6_]^−^ signal in the Ru‐C_9_ solution, we rule out the possibility of Na[PF_6_] precipitation from the solution, as both Na^+^ and [PF_6_]^−^ ions are observed in the Ru‐C_1_ solution, where the complex is not surface‐enriched – see below. Since the overall charge of the dissolved complex without counterions is −2 (considering Ru^II^ and the four negatively charged carboxylate groups), we anticipate electrostatic repulsion of negatively charged ions, including [PF_6_]^−^, from the topmost layer. We expect the anions in the underlying layers to be dominated by [OAc]^−^ due to its much larger concentration than [PF_6_]^−^. Moreover, considering the orientation of the C_9_ alkyl chains towards the vacuum, the XPS signals from the underlying layers will be strongly attenuated, and thus, any [PF_6_]^−^ present in the layers below the topmost layer would be hardly detected due to its very low overall concentration.

The Ru : N ratio calculated from the 0° spectra is 1 : 5.1 (see Table S1a), in good agreement with the expected 1 : 6 ratio, indicating that the bpy ligands are coordinated to the Ru center in solution.

Most notably, the complex‐specific Ru 3d_5/2_ and N_bpy_ signals show a much larger intensity than nominally expected even in the bulk‐sensitive 0° spectra, and with a slight increase at 80° (see Table S1). The same is true for the C_alkyl_ signal, which has a large contribution from the complex, with a much stronger increase at 80°. These observations indicate a pronounced enrichment of the complex at the IL/vacuum interface. The larger increase of the C_alkyl_ signal compared to the Ru 3d_5/2_ (see inset in Figure 2a) and N_bpy_ signals at 80° suggests that the surface is terminated with the C_9_ chains of the complex pointing towards the IL/vacuum interface, while the metal center is located below. Thus, we identify the C_9_ chains as the surface‐active moiety, which act like buoys pulling the complex to this interface. In line with the enrichment of the complex, the C_C2_, C_hetero’_ and O_OAc/COO_ signals with major contributions from the IL are smaller than nominally expected, indicative of the depletion of [C_2_C_1_Im][OAc] from the IL/vacuum interface. This depletion of the IL is most evident from the IL‐specific N_Im_ signal, which shows a low intensity at 0° and strongly decreases at 80°.

The buoy effect is not observed for the complex with C_1_ chains instead of C_9_ chains, as evident from Figure 2c (full set of XP spectra shown in Figure S3): The Ru 3d_5/2_, N_bpy_ and C_alkyl_ signals of a 1 %_mol_ Ru‐C_1_ solution clearly show a much lower intensity than those of the 1 %_mol_ Ru‐C_9_ solution. The Ru 3d and N_bpy_ signals 0° are even lower than nominally expected (Table S1b), and decrease at 80°, indicating depletion of the complex from the IL/vacuum interface. The slight increase of the C_alkyl_ and the slight decrease of the N_Im_ and O_OAc/COO_ signals at 80° stem from the preferential surface orientation of the [C_2_C_1_Im]^+^ and [OAc]^−^ ions, with the ethyl and methyl moieties pointing towards the vacuum.^[20]^ These observations reveal that the Ru‐C_1_ complex has no surface affinity and is depleted from the interface, in contrast with the buoy‐like behaviour found for the Ru‐C_9_ complex.

**Figure 3:**
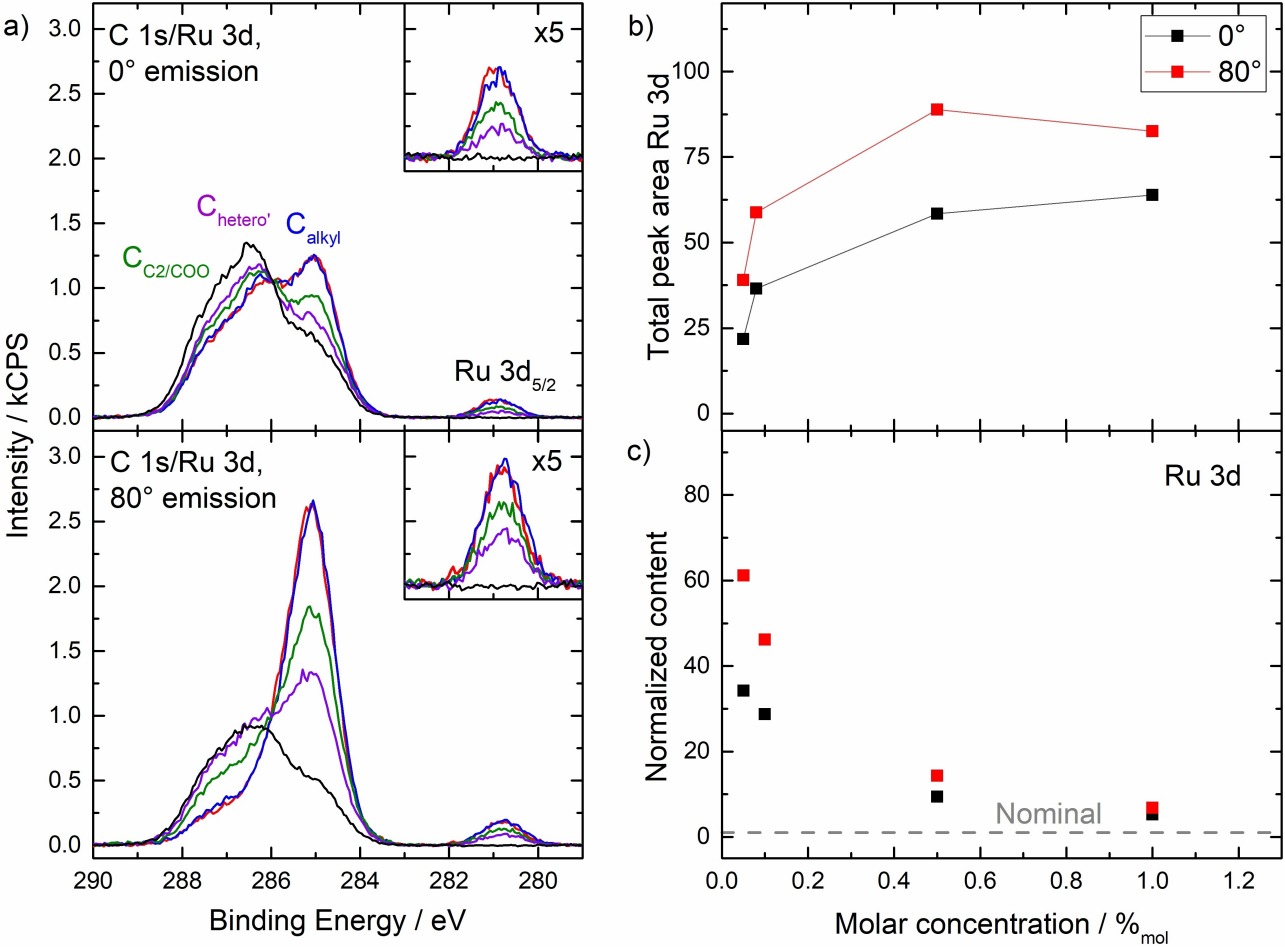
a) C 1s/Ru 3d XP spectra of solutions of Ru‐C_9_ in [C_2_C_1_Im][OAc] at 0° (top) and 80° emission (bottom) with concentrations of 1 %_mol_ (red), 0.5 %_mol_ (blue), 0.1 %_mol_ (green) and 0.05 %_mol_ (violet); upscaled Ru 3d_5/2_ signals (x5) are depicted in the insets. For comparison, we also show the spectra of neat [C_2_C_1_Im][OAc] (black). b) Absolute Intensities of Ru 3d signal and c) normalized content derived from Ru 3d signal at 0° (black) and 80° (red) over a concentration range from 1 %_mol_ to 0.05 %_mol_.

To investigate the concentration dependence, we studied Ru‐C_9_ solutions from 1 %_mol_ to 0.05 %_mol_; see Figure 3, Figures S1 and S5‐S7 and the quantitative analysis in Table S2 in the SI. While the C 1s/Ru 3d XP spectra at 0° (top) and 80° (bottom) show no significant differences at 1 %_mol_ (red) and 0.5 %_mol_ (blue), the spectra for 0.1 %_mol_ (green) and 0.05 %_mol_ (violet) gradually decrease (for comparison, also the spectra of the neat IL are shown in black). This behaviour is also evident from Figure 3b, depicting the absolute intensity of the Ru 3d signal at different concentrations. The decrease is accompanied by an increase of the C_C2/COO_ and C_hetero’_ signals at 80°. These observations imply that at concentrations of 0.5 %_mol_ and above, the IL/vacuum interface is saturated with Ru‐C_9_, while at lower concentrations no saturation is achieved. It should be noted that at saturation of the interface with the complex, the outermost surface layer must also include IL [C_2_C_1_Im]^+^ cations and potentially [OAc]^−^ anions to maintain charge neutrality, compensating for the depletion of Na^+^ and [PF_6_]^−^ ions. Additionally, the XP signal at 80° predominantly originates from the topmost layer, constituting approximately 80 % of the total signal (assuming a layer thickness of 0.7–0.8 nm) with the remaining 20 % originating from underlying layers. Consequently, IL ions from the layers underneath inevitably contribute to the signal.^[19]^ Remarkably, the concentration required to facilitate surface saturation is much lower as observed for the PFAS‐based catalyst in [C_4_C_1_Im][PF_6_].[^13d^, ^13e^] We attribute this behaviour predominantly to the higher surface tension of our hydrophilic solvent IL [C_2_C_1_Im][OAc] of 47.1 mN/m at 298 K,^[17]^ as compared to the more hydrophobic [C_4_C_1_Im][PF_6_] with its lower surface tension of 43.4 mN/m at 298 K,^[13e]^ which favours a stronger enrichment of the surface‐active complex at the IL/vacuum interface in [C_2_C_1_Im][OAc].

To quantify the surface enrichment, we plotted the normalized Ru 3d content, representing the ratio of the experimental and nominal Ru contents at 0° (black) and 80° (red) vs concentration, in Figure 3c; thereby, a value of 1 (grey dashed line) corresponds to a homogeneously distributed and randomly orientated complex. The strong increase in normalized Ru content at both emission angles at low concentrations of Ru‐C_9_, reflects the enhanced surface enrichment relative to the bulk content. The enhancement factor in the top‐most layer is ~61 at 0.05 %_mol_ as compared to ~7 at 1 %_mol_. This finding is particularly promising for catalytic applications, as it opens the door towards a most efficient atom utilization. As pointed out above, a similar surface enhancement was derived from reactive ion scattering for a Ru complex with a tri‐octylphosphine ligand dissolved in the IL [C_2_C_1_Im][Tf_2_N] (2–3 %_mol_ at the surface vs. 0.04 %_mol_ in the bulk).^[13b]^

**Figure 4:**
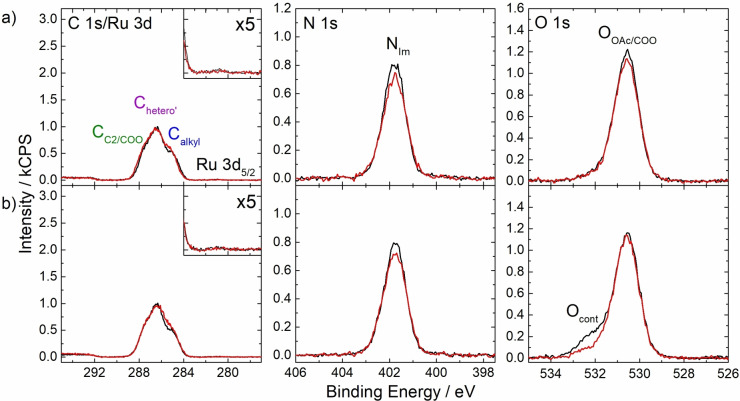
C 1s/Ru 3d, N 1s and O 1s XPS spectra regions of 1 %_mol_ solutions of a) Ru‐OC_2_ and b) Ru‐tC_4_ in [C_2_C_1_Im][OAc] at 0° (black) and 80° (red) emission. Upscaled Ru 3d_5/2_ signals (x5) are depicted in the insets. In the O 1s region of the solution of Ru‐tC_4_ a small amount of a non‐surface‐active contamination O_cont_ from the synthesis procedure was also identified which is not expected to affect the surface structure.

As a final step, we modified the length and shape of the side chains by replacing the nonyl groups with ethoxy and tert‐butyl groups (see Figure 1 for structures, and Figure S8 and S9 for full sets of XP spectra). Figures 4a and 4b show the C 1s/Ru 3d, N 1s, and O 1s spectra for 1 %_mol_ Ru‐OC_2_ and Ru‐tC_4_ solutions in [C_2_C_1_Im][OAc], with the quantitative analyses provided in Table S1c–d. Overall, the measured spectra resemble those obtained for the neat [C_2_C_1_Im][OAc], as discussed above, with the very small Ru 3d_5/2_ and N_bpy_ signals indicating a very low concentration of the Ru complex at the IL/vacuum interface. These findings imply the absence of surface enrichment of these complexes.

## Conclusions

Our goal was to evaluate different routes to tailor and quantify the surface enrichment of fluorine‐free metal complexes in SILP systems. We have successfully demonstrated that the introduction of non‐fluorinated long hydrophobic alkyl chains into the ligand system of Ru polypyridyl complexes leads to a strong surface enrichment at the IL/vacuum interface when dissolved in the hydrophilic IL [C_2_C_1_Im][OAc]. The long alkyl chains act in a buoy‐like fashion localizing the complex at the outer surface. The fluorine‐free buoy effect provides an environmentally more benign route for surface‐enriching organometallic catalysts. Ligands with shorter or bulkier groups fail to promote the accumulation of the complex at the IL surface, which underscores the buoy‐like behaviour of the long alkyl substituents. Also, the IL/vacuum interface is found to be saturated at bulk concentrations of the complex as low as of 0.5 %_mol_, which holds particular significance for catalytic applications such as in SILP, where the preferential localization of catalytically active sites at the IL/gas interface is essential for optimum atom utilization. Although the suitability of these specific complexes for the SILP concept was not shown so far, the fact that the surface enrichment is induced by the alkyl chains makes them excellent model catalysts for demonstrating this effect.

## Conflict of interests

The authors declare no conflict of interest.

1

## Supporting information

As a service to our authors and readers, this journal provides supporting information supplied by the authors. Such materials are peer reviewed and may be re‐organized for online delivery, but are not copy‐edited or typeset. Technical support issues arising from supporting information (other than missing files) should be addressed to the authors.

Supporting Information

## Data Availability

The data that support the findings of this study are available from the corresponding author upon reasonable request. Source data are provided at Zenodo: DOI: 10.5281/zenodo.11032159
