# Supplementary material for: Unlocking the Fluorine‐Free Buoy Effect: Surface‐Enriched Ruthenium Polypyridine Complexes in Ionic Liquids
Source: ChemistryOpen. 2024 Apr 30;13(7):e202400092. doi: 10.1002/open.202400092 (PMC11230926; doi:10.1002/open.202400092)
Supplement: Supplementary file 1 — Supporting Information [file OPEN-13-e202400092-s001.pdf]

ChemistryOpen

Supporting Information

# Unlocking the Fluorine-Free Buoy Effect: Surface-Enriched Ruthenium Polypyridine Complexes in Ionic Liquids

Luciano Sanchez Merlinsky, Daniel Hemmeter, Luis M. Baraldo, Florian Maier, Hans-Peter Steinrück,* and Federico J. Williams*

# Supporting Information

# Unlocking the Fluorine-Free Buoy Effect: Surface-Enriched Ruthenium Polypyridine Complexes in Ionic Liquids

Luciano Sanchez Merlinsky[a,b,#], Daniel Hemmeter[c,#], Luis M. Baraldo[a,b], Florian Maier[c], Hans-Peter Steinrück[c*] and Federico J. Williams[a,b*]

[a]Departamento de Química Inorgánica, Analítica y Química Física, Facultad de Ciencias Exactas y Naturales, Universidad de Buenos Aires, Buenos Aires, Argentina

[b]Instituto de Química Física de los Materiales, Medio Ambiente y Energía, CONICET-Universidad de Buenos Aires, Buenos Aires, Argentina

[c]Lehrstuhl für Physikalische Chemie II, Friedrich-Alexander-Universität Erlangen-Nürnberg, Egerlandstraße 3, Erlangen, Germany

[*] Corresponding author email: hans-peter.steinrueck@fau.de and fwilliams@qi.fcen.uba.ar

[#] L. Sanchez Merlinsky and D. Hemmeter contributed equally to the manuscript.

## Experimental section

$Ru(dcb)_2Cl_2$ (dcb = 4,4'-dicarboxy-2,2'-bipyridine) and 4,4'-diethoxy-2,2'-bipyridine were prepared according to previous reports.[1] 1-ethyl-3-methylimidazolium acetate ($[C_2C_1Im][OAc]$) and all other materials used in this work were obtained commercially and used without further purification.

$Ru\text{-}C_1$, $Ru\text{-}OC_2$ and $Ru\text{-}tC_4$: 50 mg of $Ru(dcb)_2Cl_2$ (0.076 mmol, 1.0 eq), 43 mg of silver triflate (0.17 mmol, 2.2 eq) and the corresponding ligand (4,4'-dimethyl-2,2'-bipyridine, 4,4'-diethoxy-2,2'-bipyridine and 4,4'-ditertbutyl-2,2'-bipyridine, respectively; 0.11 mmol, 1.5 eq) were heated at reflux covered from light for 3 h in 20 mL of ethanol. The mixture was filtered hot and its contents reduced to 5 mL. 30 mL of water was then added to precipitate the excess of ligand. After filtering the solid, the volume was reduced to 10 mL and 1 mL of $HPF_6$ (55 % wt. solution) was added, affording a dark red suspension of the protonated complex as the hexafluorophosphate salt. The complex was filtered, washed with water and dried in a vacuum desiccator overnight. Finally, the product was dissolved in 5 mL of water with 4 eq of NaOH and taken to dryness. Yield: $Ru\text{-}C_1$, 52 %; $Ru\text{-}OC_2$, 15 %; $Ru\text{-}tC_4$, 13 %. $^1H$-NMR (500 MHz, $D_2O\text{-}K_2CO_3$): $Ru\text{-}C_1$, δ

ppm 8.86 (d, 4H); 8.35 (d, 2H); 7.89 (dd, 4H); 7.66 (t, 4H); 7.54 (d, 2H); 7.20 (d, 2H); 2.51 (t, 6H). Ru-$OC_2$, δ ppm 8.86 (d, 4H); 7.98 (d, 2H); 7.88 (dd, 4H); 7.72 (dd, 2H); 7.64 (dd, 2H); 7.48 (d, 2H); 6.91 (dd, 2H); 4.23 (m, 4H); 1.39 (t, 6H). Ru-t$C_4$, δ ppm 8.82 (d, 4H); 8.39 (d, 2H); 7.85 (dd, 4H); 7.61 (m, 6H); 7.35 (dd, 2H); 1.31 (s, 18H).

Ru-$C_9$: 50 mg of $Ru(dcb)_2Cl_2$ (0.076 mmol, 1.0 eq), 43 mg of silver triflate (0.17 mmol, 2.2 eq) and 46 mg of 4,4'-dinonyl-2,2'-bipyridine (0.11 mmol, 1.5 eq) were heated at reflux covered from light for 3 h in 20 mL of ethanol. The mixture was filtered hot and its contents reduced to 5 mL. 3 mL of saturated $KPF_6$ water solution were added and then another 10 mL of water to precipitate the product as the hexafluorophosphate salt together with the excess of ligand. After filtering the solid and washing it with water, it was dissolved in 5 mL of NaOH 0.1 M, filtered and neutralized with 2 mL of HCl 0.5 M, affording a dark red suspension. The complex was filtered, washed with water and dried in a vacuum desiccator overnight. Finally, the product was dissolved in 5 mL of water with 4 eq of NaOH and taken to dryness. Yield: 63 %. $^1$H-NMR (500 MHz, $D_2O$-$K_2CO_3$): δ ppm 8.91 (dd, 2H); 8.85 (d, 2H); 8.28 (d, 2H); 7.80 (d, 2H); 7.68 (m, 8H); 7.25 (d, 2H); 2.60 (t, 4H); 1.41 (m, 4H); 0.91 (m, 24H); 0.57 (t, 6H).

Solutions of the complexes were prepared by stirring the compounds for at least 2 h under ambient conditions in the IL before introducing them into the load lock of the ultra-high vacuum (UHV) system, where the samples were left for degassing for several hours. The weighed proportions for preparation of the solutions are shown in Table S3.

ARXPS analyses were conducted using the unique Dual Analyzer System for Surface Analysis (DASSA), which is described elsewhere.[2] In brief, the setup comprises two analyzers at 0° (normal emission) and 80° (grazing) emission angle allowing for simultaneous recording of XP spectra in these geometries. Using Al Kα radiation, at 0° the information depth (ID) in organic matter is 6-9 nm and the spectra are dominated by bulk contributions; at 80°, the ID is only 1-1.5 nm and the spectra are dominated by to the first molecular layer of the sample. The intensity detected in the XP spectra was normalized to the overall intensity (sum over all intensities corrected by the atomic sensitivity factors (ASF)[3] of the $0.1\%_{mol}$ solution of Ru-$C_9$ at 0°. Further information on treatment and fitting of the ARXP spectra can be found elsewhere.[4]

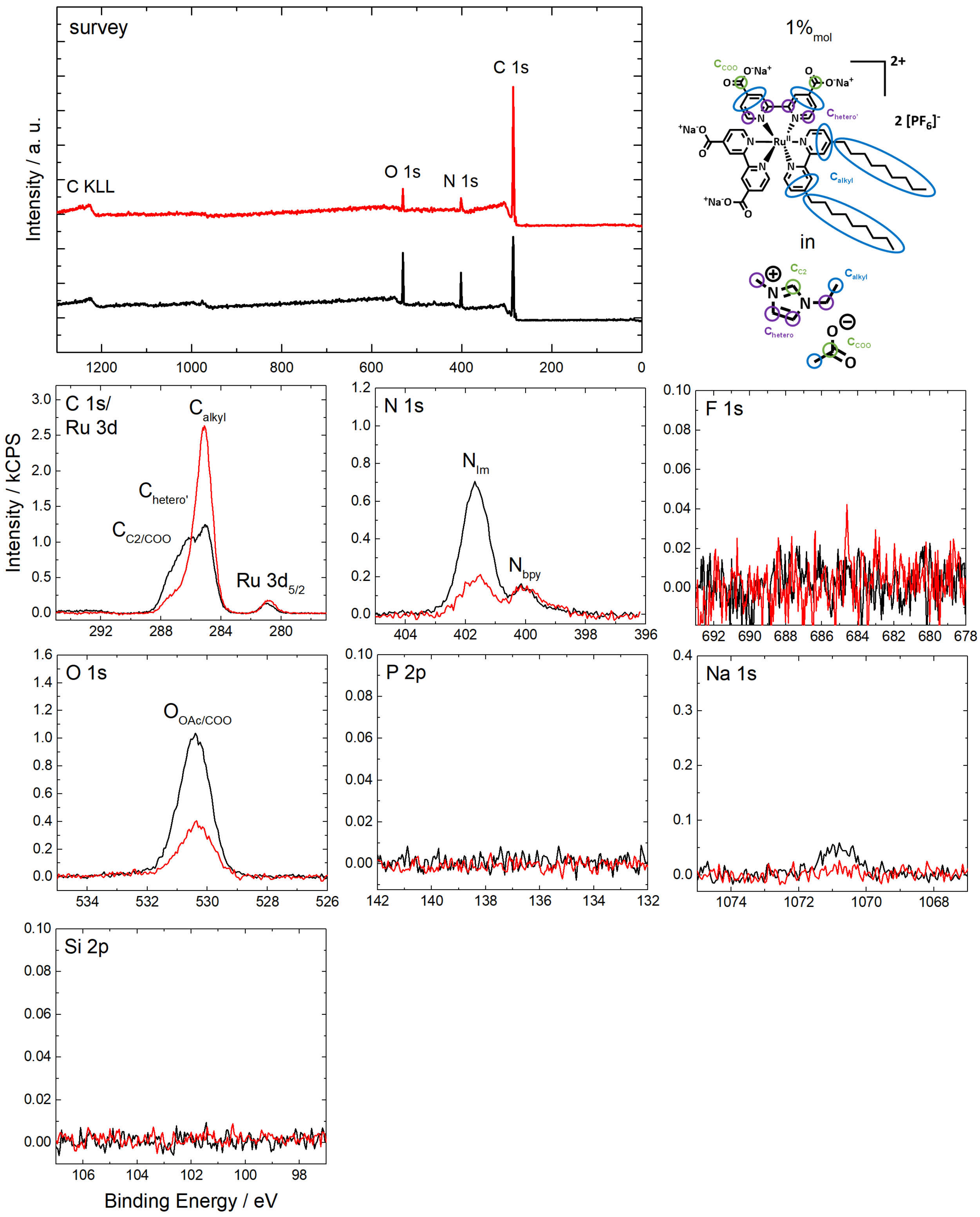


***Figure S1***: *Survey, C 1s/Ru 3d, N 1s, F 1s, O 1s, P 2p, Na 1s and Si 2p XP spectra of 1%$_{mol}$ Ru-C$_9$ in [C$_2$C$_1$Im][OAc] in 0° (black) and 80° (red) emission recorded at room temperature. The Si 2p spectra are shown to confirm absence of common surface-active contaminations.*[5]

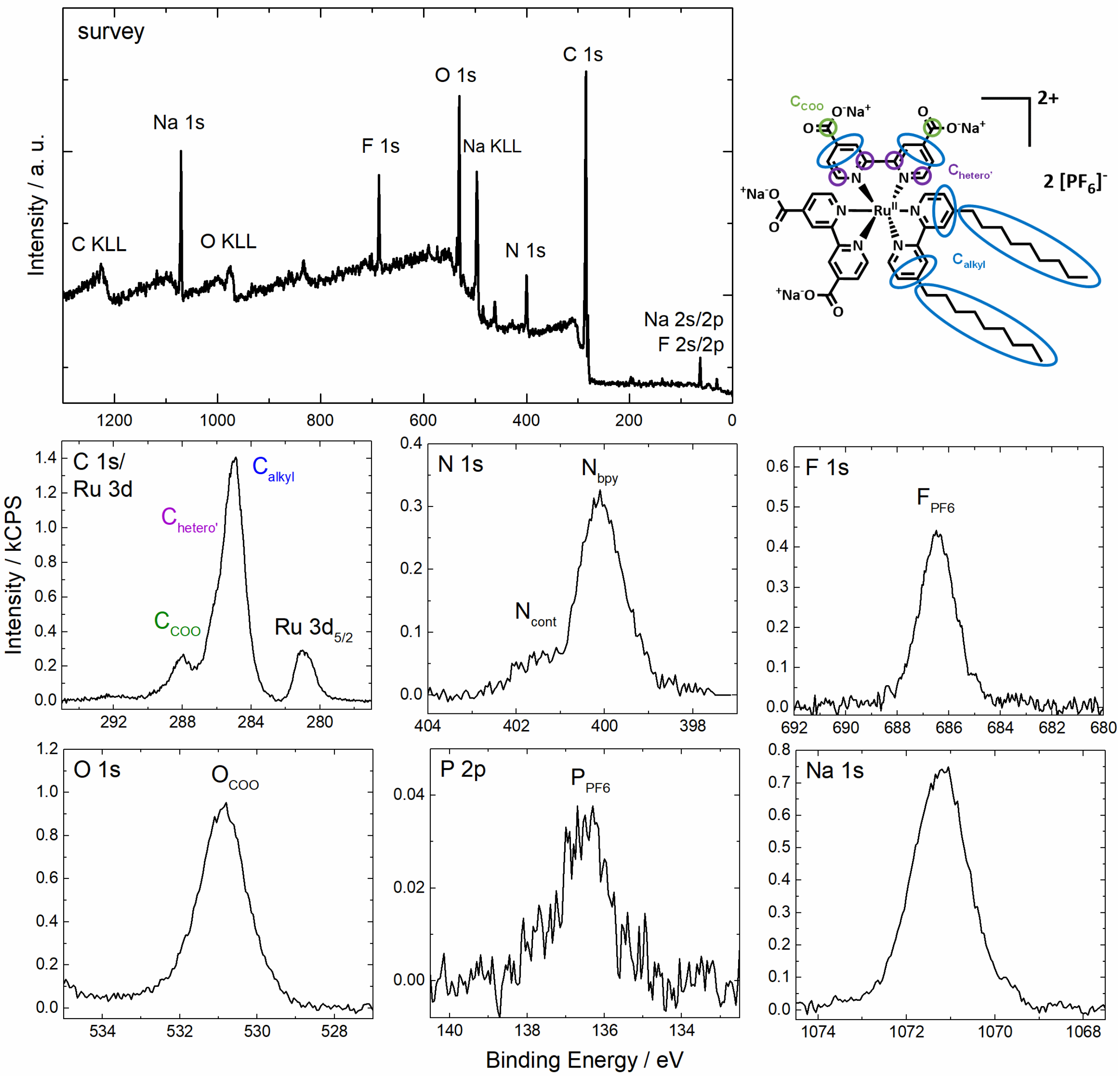


***Figure S2***: *Survey, C 1s/Ru 3d, N 1s, F 1s, O 1s, P 2p and Na 1s XP spectra of solid Ru-C₉ in 0° emission recorded at room temperature. Nominal Ru:N:Na ratio for Ru-C₉: 1:6:4. Found: 1:5.3:3.5. A small amount of contaminations from the synthesis procedure is also identified at the N 1s region. Due to sample charging, all spectra are referenced to the $C_{alkyl}$ signal, which was set to 285 eV as in solution.*

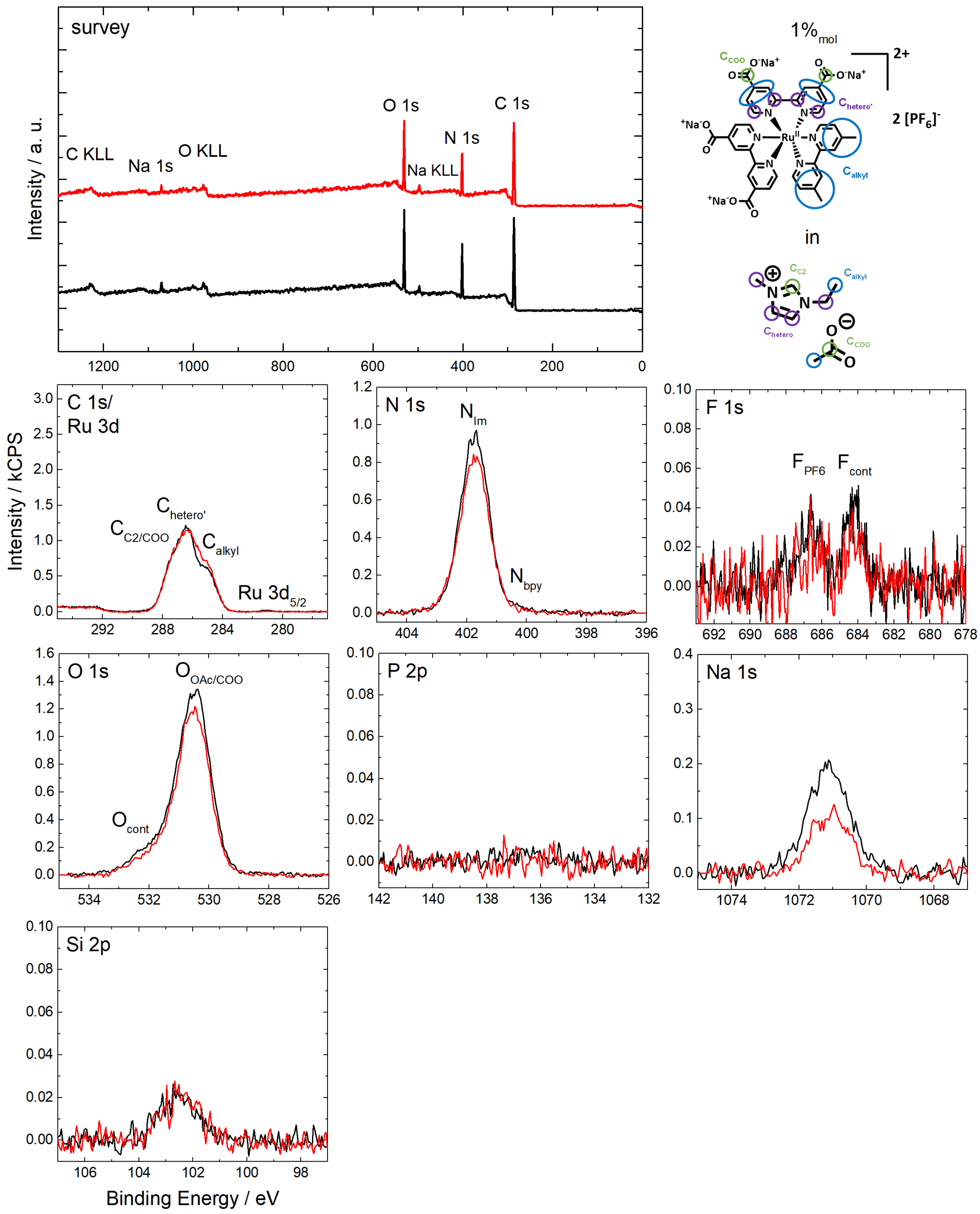


***Figure S3**: Survey, C 1s/Ru 3d, N 1s, F 1s, O 1s, P 2p, Na 1s and Si 2p XP spectra of 1%$_{mol}$ Ru-$C_1$ in [$C_2C_1$Im][OAc] in 0° (black) and 80° (red) emission recorded at room temperature. A small amount of non-surface-active contaminations from the synthesis procedure is also identified at the F 1s, O 1s and Si 2p regions.*

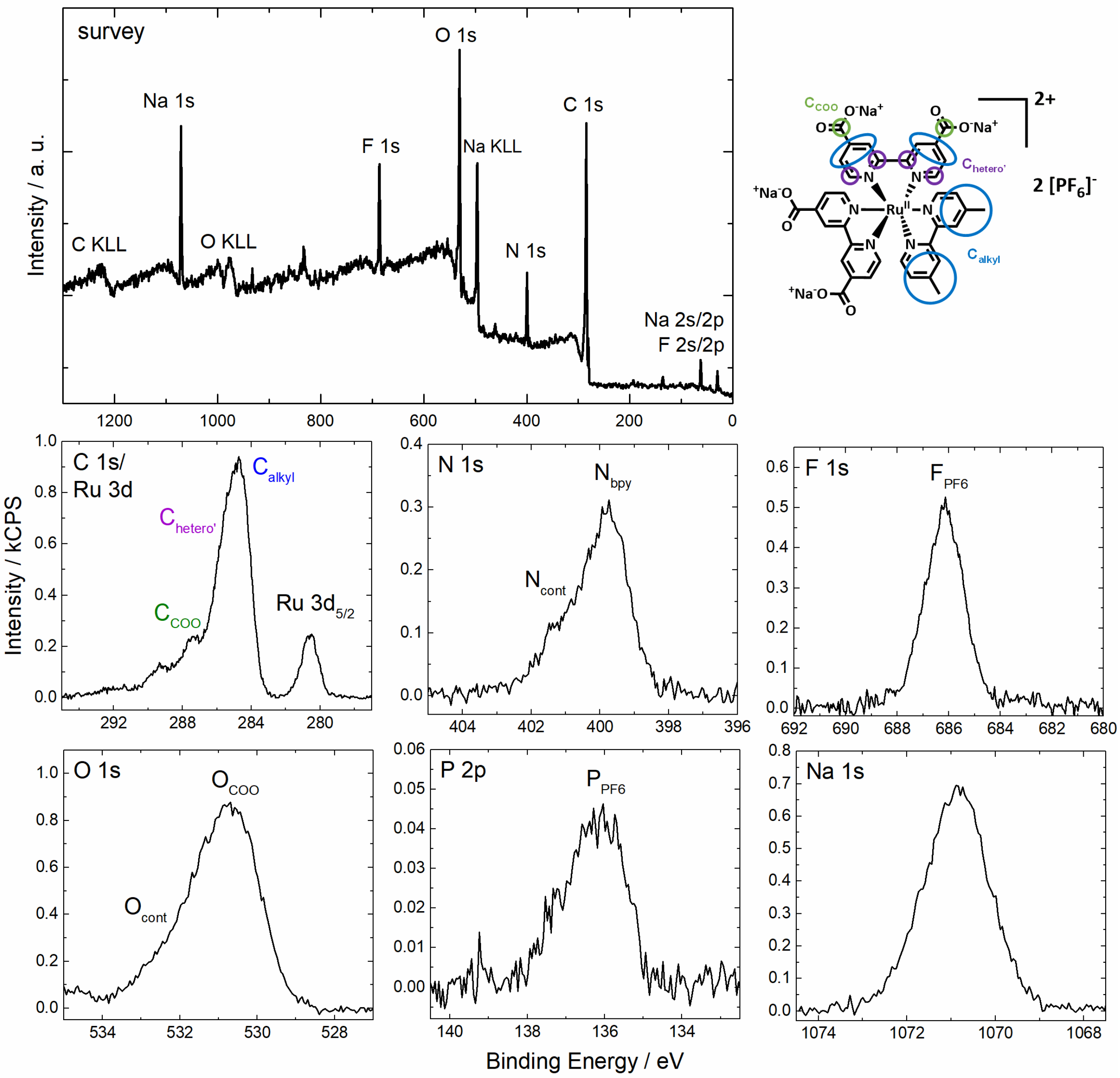


***Figure S4**: Survey, C 1s/Ru 3d, N 1s, F 1s, O 1s, P 2p and Na 1s XP spectra of solid Ru-$C_1$ in 0° emission recorded at room temperature. Nominal Ru:N:Na ratio for Ru-$C_1$: 1:6:4. Found: 1:5.7:3.7. A small amount of contaminations from the synthesis procedure is also identified at the N 1s and O 1s regions. Due to sample charging, all spectra are referenced to the $C_{alkyl}$ signal, which was set to 285 eV as in solution.*

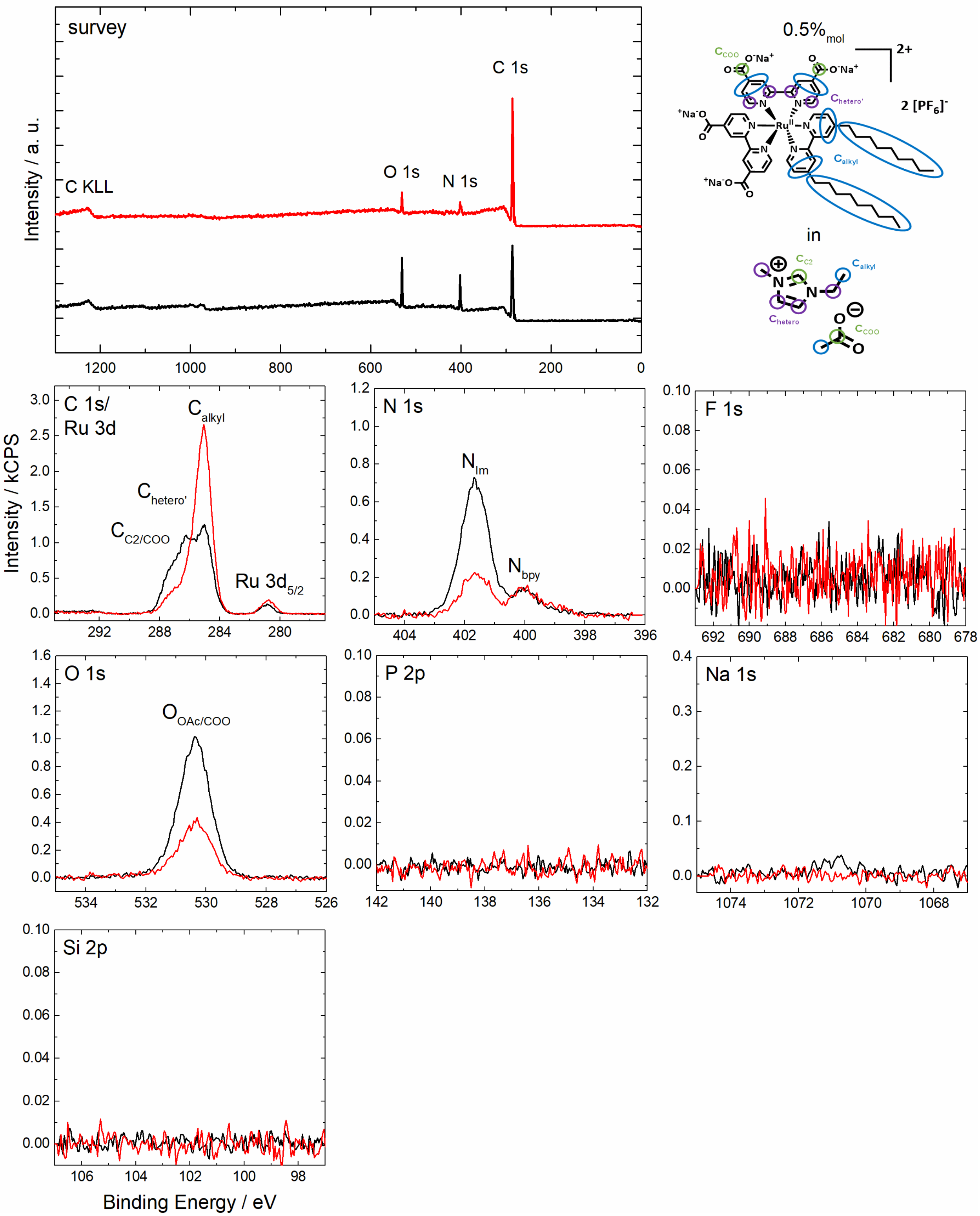


***Figure S5**: Survey, C 1s/Ru 3d, N 1s, F 1s, O 1s, P 2p, Na 1s and Si 2p XP spectra of 0.5%$_{mol}$ Ru-C$_9$ in [C$_2$C$_1$Im][OAc] in 0° (black) and 80° (red) emission recorded at room temperature.*

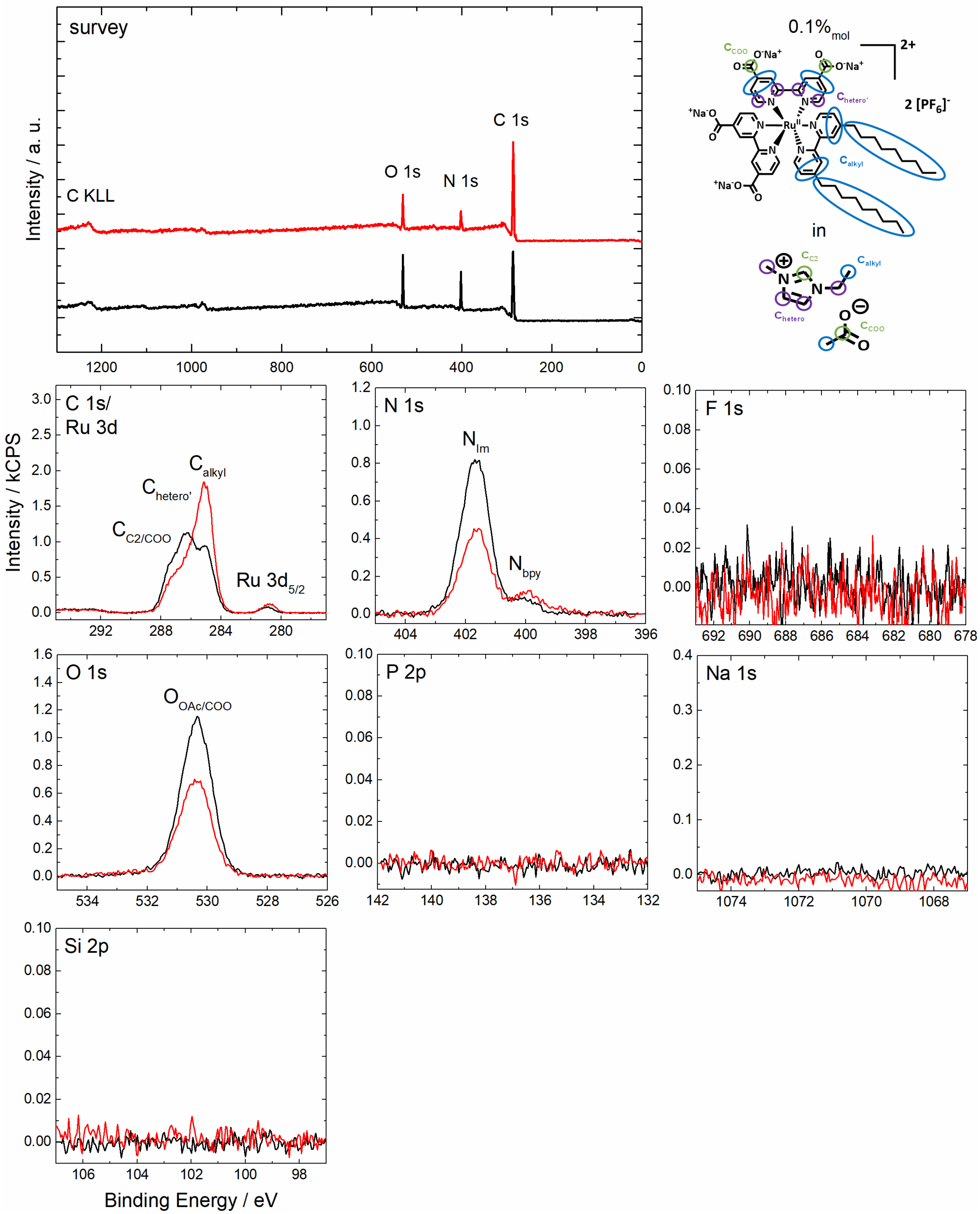


***Figure S6**: Survey, C 1s/Ru 3d, N 1s, F 1s, O 1s, P 2p, Na 1s and Si 2p XP spectra of 0.1%$_{mol}$ Ru-C$_9$ in [C$_2$C$_1$Im][OAc] in 0° (black) and 80° (red) emission recorded at room temperature.*

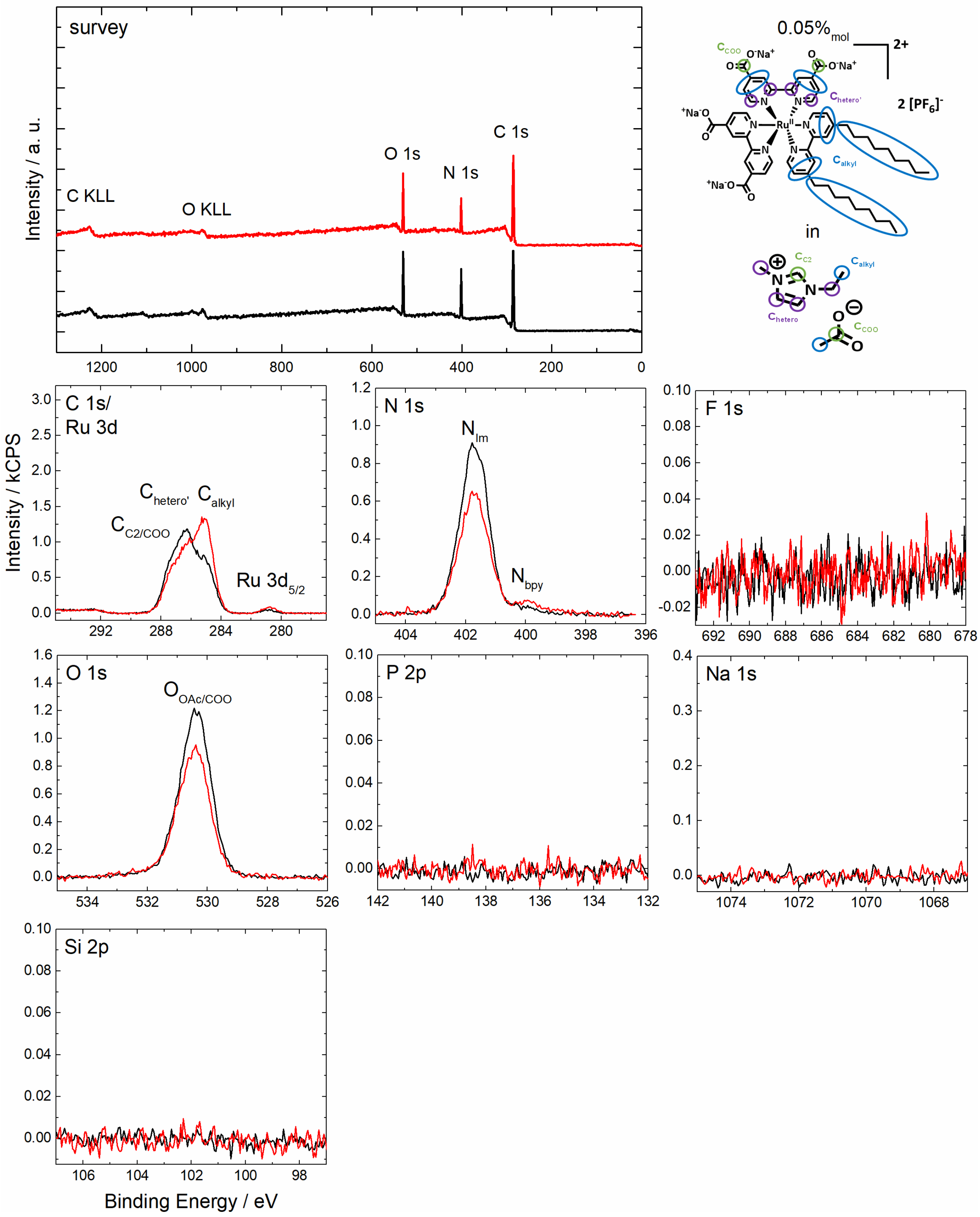


***Figure S7**: Survey, C 1s/Ru 3d, N 1s, F 1s, O 1s, P 2p, Na 1s and Si 2p XP spectra of 0.05%$_{mol}$ Ru-C$_9$ in [$C_2C_1$Im][OAc] in 0° (black) and 80° (red) emission recorded at room temperature.*

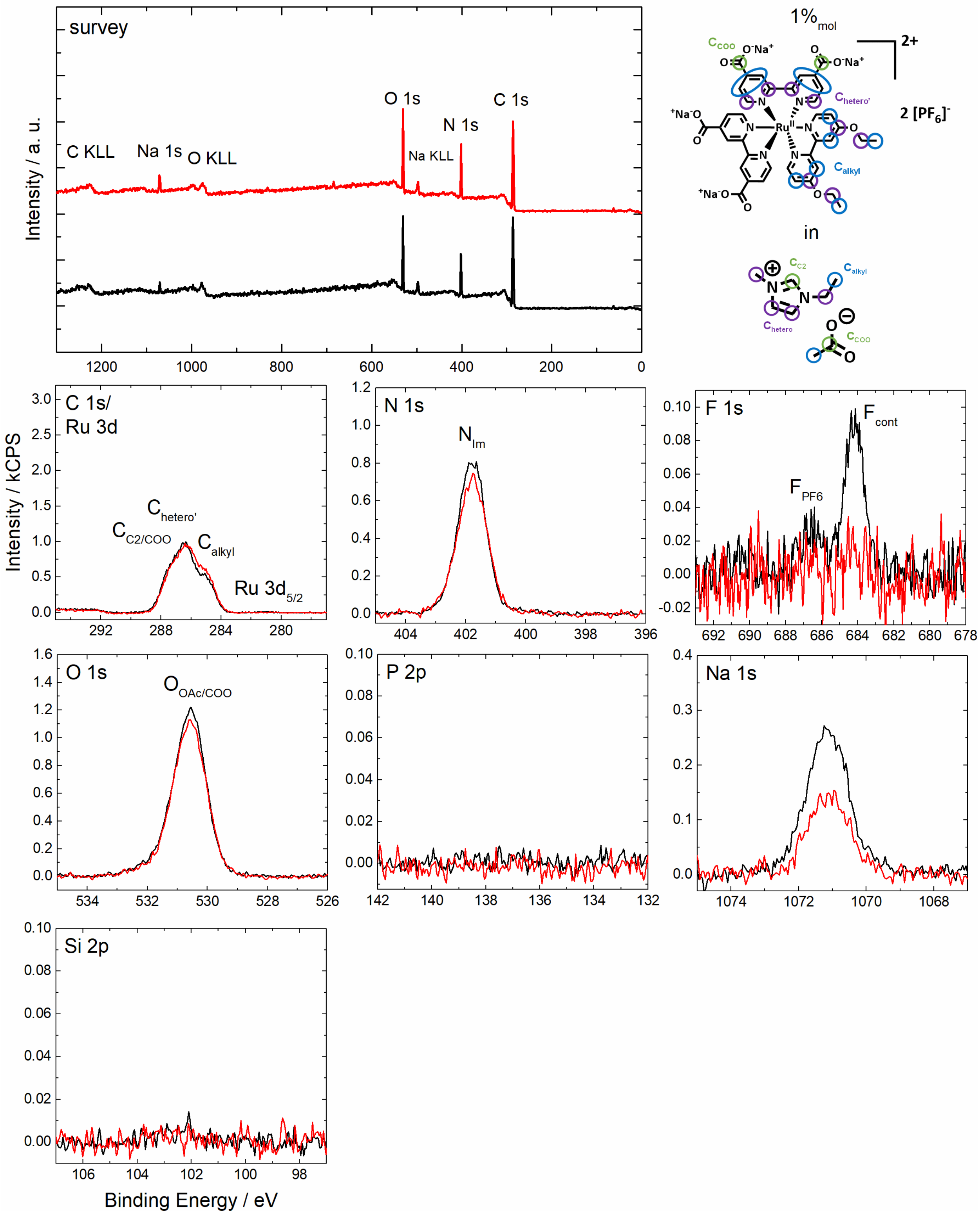


***Figure S8***: *Survey, C 1s/Ru 3d, N 1s, F 1s, O 1s, P 2p, Na 1s and Si 2p XP spectra of 1%$_{mol}$ Ru-OC$_2$ in [C$_2$C$_1$Im][OAc] in 0° (black) and 80° (red) emission recorded at room temperature. A small amount of non-surface-active contaminations from the synthesis procedure is also identified at the F 1s region which is not expected to affect the surface structure.*

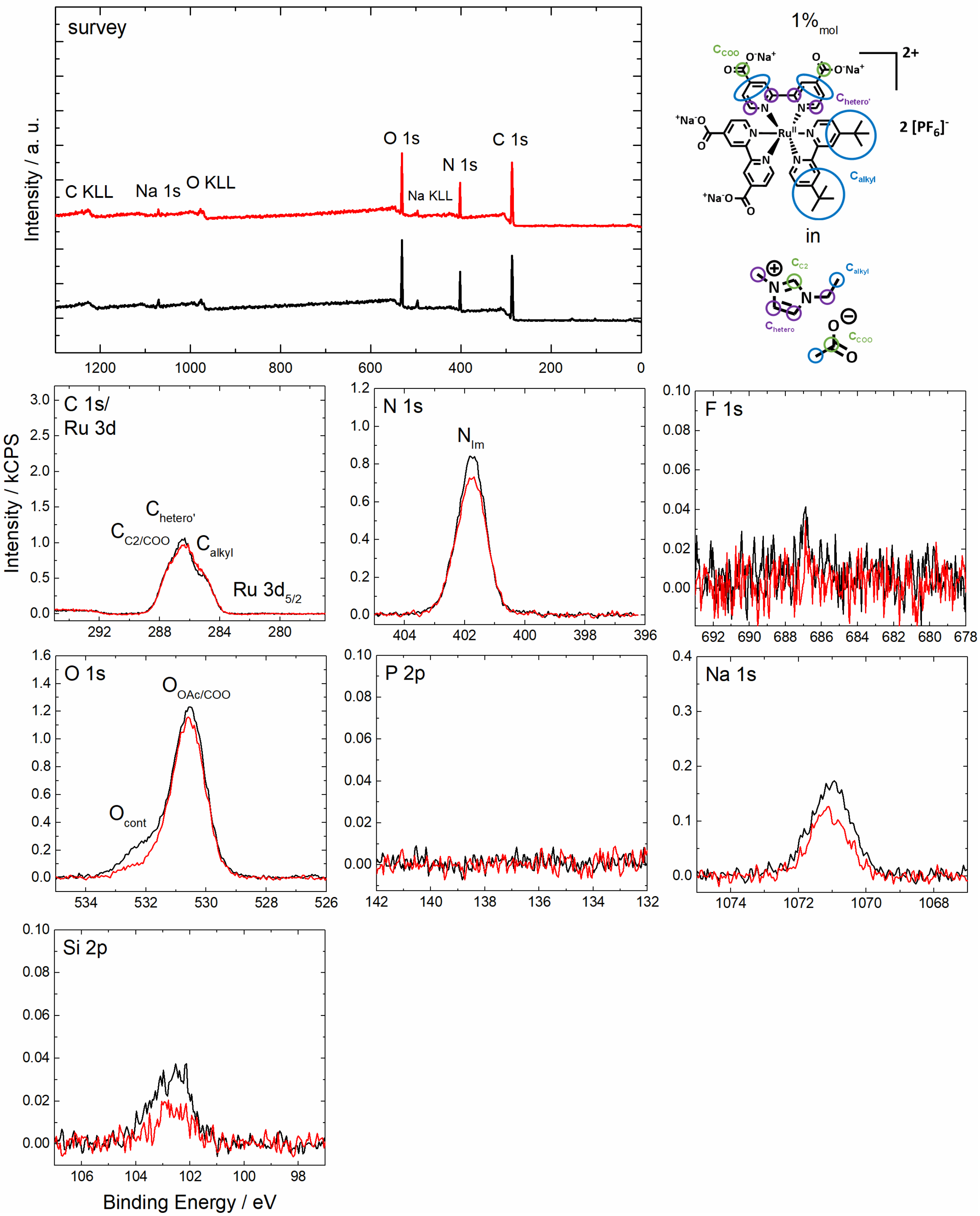


***Figure S9**: Survey, C 1s/Ru 3d, N 1s, F 1s, O 1s, P 2p, Na 1s and Si 2p XP spectra of 1%$_{mol}$ Ru-tC$_4$ in [C$_2$C$_1$Im][OAc] in 0° (black) and 80° (red) emission recorded at room temperature. A small amount of non-surface-active contaminations from the synthesis procedure is also identified at the O 1s and Si 2p regions which is not expected to affect the surface structure.*

***Table S1**. Quantitative analysis of XPS core level spectra of 1%$_{mol}$ solutions employed in this work: a) Ru-C$_9$, b) Ru-C$_1$, c) Ru-OC$_2$, d) Ru-tC$_4$ in [C$_2$C$_1$Im]OAc]. Note that the binding energies of spin-orbit-resolved signals correspond to the more intense signal at lower binding energy, that is, Ru 3d$_{5/2}$ and P 2p$_{3/2}$.*

| **a) 1%$_{mol}$ Ru-C$_9$ in [C$_2$C$_1$Im][OAc]** | **Ru 3d** | **C 1s C$_2$/COO** | **C 1s hetero** | **C 1s alkyl** | **N 1s Im** | **N 1s ligand** | **O 1s OC$_2$** | **O 1s OAc** | **F 1s** | **P 2p** | **Na 1s** |
|---|---|---|---|---|---|---|---|---|---|---|---|
| Binding Energy / eV | 280.9 | 287.4 | 286.3 | 285.0 | 401.7 | 400.0 | | 530.4 | | | 1070.8 |
| Nominal | 0.010 | 2.0 | 4.1 | 2.4 | 2.0 | 0.061 | | 2.1 | 0.12 | 0.020 | 0.040 |
| Experimental, 0° | 0.054 | 1.8 | 3.5 | 4.1 | 1.5 | 0.27 | | 1.7 | | | 0.027 |
| Experimental, 80° | 0.069 | 0.82 | 1.6 | 8.8 | 0.42 | 0.41 | | 0.67 | | | |
| **b) 1%$_{mol}$ Ru-C$_1$ in [C$_2$C$_1$Im][OAc]** | | | | | | | | | | | |
| Binding Energy / eV | 281.1 | 287.5 | 286.4 | 285.0 | 401.7 | 400.1 | | 530.5 | 686.7 | | 1071.1 |
| Nominal | 0.010 | 2.0 | 4.1 | 2.2 | 2.0 | 0.061 | | 2.1 | 0.12 | 0.020 | 0.040 |
| Experimental, 0° | 0.006 | 2.2 | 4.3 | 2.1 | 2.0 | 0.036 | | 1.9 | 0.046 | | 0.12 |
| Experimental, 80° | 0.004 | 2.2 | 4.3 | 2.5 | 1.9 | | | 1.8 | 0.029 | | 0.065 |
| **c) 1%$_{mol}$ Ru-OC$_2$ [C$_2$C$_1$Im][OAc]** | | | | | | | | | | | |
| Binding Energy / eV | 281.0 | 287.5 | 286.4 | 285.0 | 401.8 | | 531.8 | 530.6 | 686.6 | | 1071.1 |
| Nominal | 0.010 | 2.0 | 4.2 | 2.2 | 2.0 | 0.061 | 0.020 | 2.1 | 0.12 | 0.020 | 0.040 |
| Experimental, 0° | 0.005 | 2.0 | 4.1 | 2.0 | 2.0 | | 0.19 | 2.2 | 0.043 | | 0.189 |
| Experimental, 80° | | 2.0 | 4.1 | 2.4 | 1.8 | | 0.11 | 2.2 | | | 0.11 |
| **d) 1%$_{mol}$ Ru-tBu [C$_2$C$_1$Im][OAc]** | | | | | | | | | | | |
| Binding Energy / eV | 281.3 | 287.5 | 286.4 | 285.0 | 401.8 | | | 530.5 | | | 1071.0 |
| Nominal | 0.010 | 2.0 | 4.1 | 2.3 | 2.0 | 0.060 | | 2.1 | 0.12 | 0.020 | 0.040 |
| Experimental, 0° | 0.006 | 2.2 | 4.3 | 2.0 | 2.0 | | | 2.2 | | | 0.12 |
| Experimental, 80° | | 2.1 | 4.2 | 2.3 | 1.9 | | | 2.2 | | | 0.077 |

***Table S2:*** *Quantitative analysis of XPS core level spectra of solutions of Ru-$C_9$ in $[C_2C_1Im][OAc]$ with catalyst concentrations of a) $1\%_{mol}$, b) $0.5\%_{mol}$, c) $0.1\%_{mol}$ and d) $0.05\%_{mol}$.*

| **a) $1\%_{mol}$ Ru-$C_9$ in $[C_2C_1Im][OAc]$** | **Ru 3d** | **C 1s $C_2$/COO** | **C 1s hetero** | **C 1s alkyl** | **N 1s Im** | **N 1s ligand** | **O 1s OAc** | **F 1s** | **P 2p** | **Na 1s** |
|---|---|---|---|---|---|---|---|---|---|---|
| Binding Energy / eV | 280.9 | 287.4 | 286.3 | 285.0 | 401.7 | 400.0 | 530.4 | | | 1070.8 |
| Nominal | 0.010 | 2.0 | 4.1 | 2.4 | 2.0 | 0.061 | 2.1 | 0.12 | 0.020 | 0.040 |
| Experimental, 0° | 0.054 | 1.8 | 3.5 | 4.1 | 1.5 | 0.27 | 1.7 | | | 0.027 |
| Experimental, 80° | 0.069 | 0.82 | 1.6 | 8.8 | 0.42 | 0.41 | 0.67 | | | |
| **b) $0.5\%_{mol}$ Ru-$C_9$ in $[C_2C_1Im][OAc]$** | | | | | | | | | | |
| Binding Energy / eV | 280.9 | 287.4 | 286.3 | 285.0 | 401.7 | 400.0 | 530.4 | | | 1070.9 |
| Nominal | 0.005 | 2.0 | 4.1 | 2.2 | 2.0 | 0.030 | 2.0 | 0.060 | 0.010 | 0.020 |
| Experimental, 0° | 0.047 | 1.7 | 3.5 | 3.8 | 1.4 | 0.28 | 1.6 | | | 0.016 |
| Experimental, 80° | 0.072 | 0.88 | 1.8 | 8.2 | 0.43 | 0.36 | 0.68 | | | |
| **c) $0.1\%_{mol}$ Ru-$C_9$ in $[C_2C_1Im][OAc]$** | | | | | | | | | | |
| Binding Energy / eV | 280.9 | 287.4 | 286.3 | 285.0 | 401.7 | 400.0 | 530.4 | | | |
| Nominal | 0.001 | 2.0 | 4.0 | 2.0 | 2.0 | 0.006 | 2.0 | 0.012 | 0.002 | 0.004 |
| Experimental, 0° | 0.029 | 1.9 | 3.7 | 2.9 | 1.7 | 0.12 | 1.7 | | | |
| Experimental, 80° | 0.046 | 1.2 | 2.5 | 6.0 | 0.94 | 0.25 | 1.1 | | | |
| **d) $0.05\%_{mol}$ Ru-$C_9$ in $[C_2C_1Im][OAc]$** | | | | | | | | | | |
| Binding Energy / eV | 280.9 | 287.4 | 286.3 | 285.0 | 401.7 | 400.0 | 530.4 | | | |
| Nominal | 0.0005 | 2.0 | 4.0 | 2.0 | 2.0 | 0.003 | 2.0 | 0.006 | 0.001 | 0.002 |
| Experimental, 0° | 0.017 | 2.0 | 3.9 | 2.4 | 1.8 | 0.060 | 1.8 | | | |
| Experimental, 80° | 0.031 | 1.5 | 3.1 | 4.5 | 1.4 | 0.14 | 1.5 | | | |

*Table S3: Weighed proportions for preparation of the $[C_2C_1Im][OAc]$ solutions investigated in this work.*

| | 1%$_{mol}$ Ru-C$_9$ | 1%$_{mol}$ Ru-C$_1$ | 0.5%$_{mol}$ Ru-C$_9$ | 0.1%$_{mol}$ Ru-C$_9$ | 0.05%$_{mol}$ Ru-C$_9$ | 1%$_{mol}$ Ru-OC$_2$ | 1%$_{mol}$ Ru-tC$_4$ |
|---|---|---|---|---|---|---|---|
| **Mass complex / mg** | 34.8 | 10.4 | 8.4 | | 10.0 | 7.9 | 7.2 |
| **Amount of substance complex / mmol** | 0.025 | 0.009 | 0.006 | 0.003 | 0.007 | 0.007 | 0.006 |
| **Mass $[C_2C_1Im][OAc]$ / mg** | 434.7 | 155.0 | 211.6 | 538.1 | 2523 | 111.8 | 100.9 |
| **Amount of substance $[C_2C_1Im][OAc]$ / mmol** | 2.50 | 0.892 | 1.22 | 3.10 | 14.5 | 0.644 | 0.581 |
| **Mass 0.5%$_{mol}$ solution Ru-C$_9$ in $[C_2C_1Im][OAc]$ / mg** | | | | 111.4 | | | |
| **Exact molar concentration of complex in IL / %$_{mol}$** | 1.00 | 1.00 | 0.50 | 0.10 | 0.050 | 1.00 | 1.03 |
| **Exact ratio IL:complex$_l$** | 99.0:1 | 99.0:1 | 199.0:1 | 999.0:1 | 1999:0 | 99.0:1 | 99.7:1 |

$M_{[Ru\text{-}C_9]}$ = 1376.01 g/mol, $M_{[Ru\text{-}C_1]}$ = 1151.58 g/mol, $M_{[Ru\text{-}OC_2]}$ = 1211.63 g/mol,

$M_{[Ru\text{-}tC_4]}$ = 1235.74 g/mol, $M_{[C_2C_1Im][OAc]}$ = 170.21 g/mol

Purity $[C_2C_1Im][OAc]$: 98%

## References


[1] a) G. Maerker, F. H. Case, *J. Am. Chem. Soc.* **1958**, *80*, 2745-2748; b) M. Zhou, G. P. Robertson, J. Roovers, *Inorg. Chem.* **2005**, *44*, 8317-8325.

[2] I. Niedermaier, C. Kolbeck, H.-P. Steinrück, F. Maier, *Rev. Sci. Instrum.* **2016**, *87*, 045105.

[3] C. D. Wagner, L. E. Davis, M. V. Zeller, J. A. Taylor, R. H. Raymond, L. H. Gale, *Surf. Interface Anal.* **1981**, *3*, 211-225.

[4] D. Hemmeter, L. Sanchez Merlinsky, L. M. Baraldo, F. Maier, F. J. Williams, H.-P. Steinrück, *Phys. Chem. Chem. Phys.* **2023**, submitted.

[5] J. M. Gottfried, F. Maier, J. Rossa, D. Gerhard, P. S. Schulz, P. Wasserscheid, H.-P. Steinrück, *Z. Phys. Chem.* **2006**, *220*, 1439-1453.
